# Correction: The scavenger receptor repertoire in six cnidarian species and its putative role in cnidarian-dinoflagellate symbiosis

**DOI:** 10.7717/peerj.2692/correction-1

**Published:** 2017-06-06

**Authors:** Emilie F. Neubauer, Angela Z. Poole, Olivier Detournay, Virginia M. Weis, Simon K. Davy

**Affiliations:** 1 School of Biological Sciences, Victoria University of Wellington, Wellington, New Zealand; 2 Department of Biology, Western Oregon University, Monmouth, OR, United States; 3 Department of Integrative Biology, Oregon State University, Corvallis, OR, United States; 4 PLANKTOVIE sas, Allauch, France

The author list should have included Olivier Detournay as the 3rd author in the author list. He is also the cofounder of Planktovie. He was mistakenly omitted from the author list during the review process.

