# Supplementary material for: The scavenger receptor repertoire in six cnidarian species and its putative role in cnidarian-dinoflagellate symbiosis
Source: PeerJ. 2016 Nov 15;4:e2692. doi: 10.7717/peerj.2692 (PMC5119243; doi:10.7717/peerj.2692)
Supplement: Supplemental Information 1 [file peerj-04-2692-s001.pdf]

Table S1. Metazoan resources searched to identify sequences for this study.

Phylum	Organism	Resource	Reference
Ctenophora	*Mnemiopsis leidyi*	http://research.nhgri.nih.gov/mnemiopsis/blast/	(Ryan et al. 2013)
Porifera	*Oscarella carmella*	http://www.compagen.org/index.html	(Nichols et al. 2012)
	Suberites domuncula	NCBI: http://www.ncbi.nlm.nih.gov/	(Harcet et al. 2010)
	Amphimedon queenslandica	http://metazoa.ensembl.org/Amphimedon_queenslandica	(Srivastava et al. 2010)
Placozoa	*Trichoplax adhaerens*	http://genome.jgi-psf.org/Triad1/Triad1.home.html	(Srivastava et al. 2008)
Cnidaria	*Nematostella vectensis*	http://genome.jgi-psf.org/Nemve1/Nemve1.home.html	(Putnam et al. 2007)
	Anthopleura elegantissima	http://people.oregonstate.edu/~meyere/data.html	(Kitchen et al. 2015)
	Aiptasia pallida	http://pringlelab.stanford.edu/projects.html	(Lehnert et al. 2012)<br>(Baumgarten et al. 2015)
	Acropora digitifera	http://marinegenomics.oist.jp/genomes/gallery	(Shinzato et al. 2011)
	Acropora millepora	http://www.bio.utexas.edu/research/matz_lab/matzlab/Data.html	(Moya et al. 2012)
	Fungia scutaria	http://people.oregonstate.edu/~meyere/data.html	(Kitchen et al. 2015)
Nemotoda	*Caenorhabditis elegans*	http://www.wormbase.org/	(Stein et al. 2001)
Arthropoda	*Drosophila melanogaster*	NCBI: http://www.ncbi.nlm.nih.gov/	
Echinodermata	*Strongylocentrotus purpuratus*	http://www.spbase.org/SpBase/	(Cameron et al. 2009)
Chordata	*Ciona intestinalis*	http://genome.jgi-psf.org/Cioin2/Cioin2.home.html	(Dehal et al. 2002)
	Branchiostoma floridae	http://genome.jgi- psf.org/Brafl1/Brafl1.home.html	(Putnam et al. 2008)
	Xenopus laevis	NCBI: http://www.ncbi.nlm.nih.gov/	(Klein et al. 2002)
	Danio rerio	NCBI: http://www.ncbi.nlm.nih.gov/	
	Mus musculus	NCBI: http://www.ncbi.nlm.nih.gov/	
	Homo sapiens	NCBI: http://www.ncbi.nlm.nih.gov/	

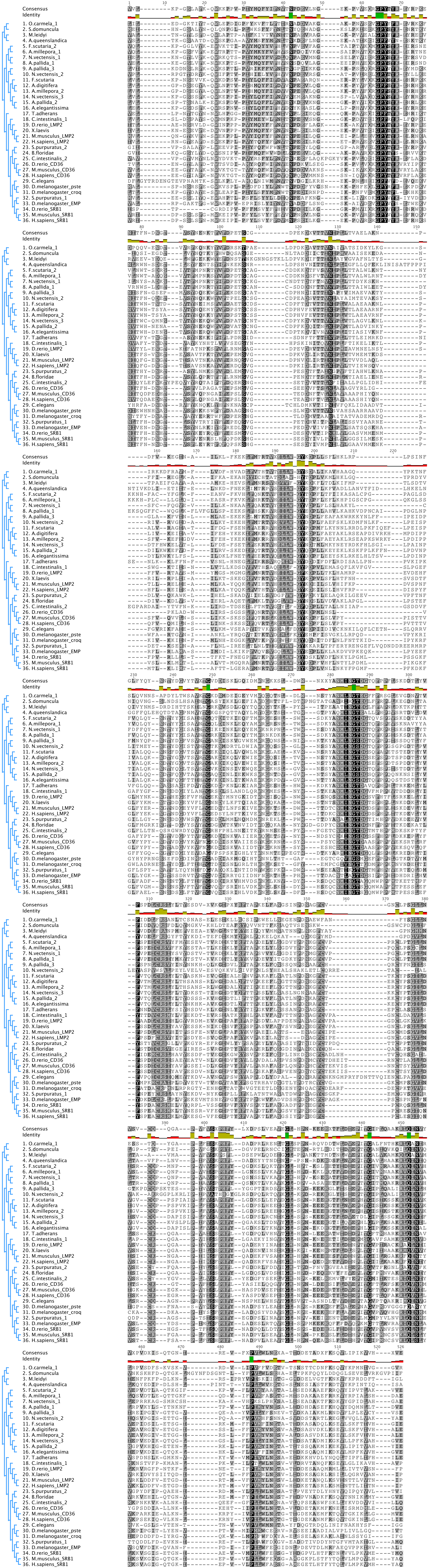
Consensus
Identity
1. O.carmela_1
2. S.domuncula
3. M.leidyi
4. A.queenslandica
5. F.scutaria_2
6. A.millepora_1
7. N.vectensis_1
8. A.pallida_1
9. A.pallida_3
10. N.vectensis_2
11. F.scutaria
12. A.digitifera
13. A.millepora_2
14. N.vectensis_3
15. A.pallida_2
16. A.elegantissima
17. T.adherans
18. C.intestinalis_1
19. D.rerio_LMP2
20. X.laevis
21. M.musculus_LMP2
22. H.sapiens_LMP2
23. S.purpuratus_2
24. B.floridae
25. C.intestinalis_2
26. D.rerio_CD36
27. M.musculus_CD36
28. H.sapiens_CD36
29. C.elegans
30. D.melanogaster_pste
31. D.melanogaster_croq
32. S.purpuratus_1
33. D.melanogaster_EMP
34. D.rerio_SRB1
35. M.musculus_SRB1
36. H.sapiens_SRB1

Figure S1. Multiple sequence alignment of the CD36 domain from SR-B homologues of cnidarians and select other metazoans. Cnidarian proteins lack one of the three pairs of cysteine residues known to form three disulphide bridges in the human CD36 protein. All six cnidarian species have a pair of cysteine residues at positions C107 and C117.

References

Baumgarten S, Simakov O, Esherick LY, Liew YJ, Lehnert EM, Michell CT, Li Y, Hambleton EA, Guse A, Oates ME, Gough J, Weis VM, Aranda M, Pringle JR, and Voolstra CR. 2015. The genome of *Aiptasia*, a sea anemone model for coral symbiosis. *Proceedings of the National Academy of Sciences* 112:11893-11898. DOI: 10.1073/pnas.1513318112

Cameron RA, Samanta M, Yuan A, He D, and Davidson E. 2009. SpBase: the sea urchin genome database and web site. *Nucleic Acids Research* 37:D750-D754. DOI: 10.1093/nar/gkn887

Dehal P, Satou Y, Campbell RK, Chapman J, Degnan B, De Tomaso A, Davidson B, Di Gregorio A, Gelpke M, and Goodstein DM. 2002. The draft genome of *Ciona intestinalis*: insights into chordate and vertebrate origins. *Science* 298:2157-2167. DOI: 10.1126/science.1080049

Harcet M, Roller M, Ćetković H, Perina D, Wiens M, Müller WEG, and Vlahoviček K. 2010. Demosponge EST sequencing reveals a complex genetic toolkit of the simplest metazoans. *Molecular Biology and Evolution* 27:2747-2756. DOI: 10.1093/molbev/msq174

Kitchen SA, Crowder CM, Poole AZ, Weis VM, and Meyer E. 2015. *De novo* assembly and characterization of four anthozoan (Phylum Cnidaria) transcriptomes. *G3: Genes|Genomes|Genetics* 5:2441-2452. DOI: 10.1534/g3.115.020164

Klein SL, Strausberg RL, Wagner L, Pontius J, Clifton SW, and Richardson P. 2002. Genetic and genomic tools for *Xenopus* research: The NIH *Xenopus* initiative. *Developmental Dynamics* 225:384-391. DOI: 10.1002/dvdy.10174

Lehnert E, Burriesci M, and Pringle J. 2012. Developing the anemone *Aiptasia* as a tractable model for cnidarian-dinoflagellate symbiosis: the transcriptome of aposymbiotic *A. pallida*. *BMC Genomics* 13:271. DOI: 10.1186/1471-2164-13-271

Moya A, Huisman L, Ball E, Hayward D, Grasso L, Chua C, Woo H, GATTUSO JP, Forêt S, and Miller D. 2012. Whole transcriptome analysis of the coral *Acropora millepora* reveals complex responses to CO_2-driven acidification during the initiation of calcification. *Molecular Ecology* 21:2440-2454. DOI: 10.1111/j.1365-294X.2012.05554.x

Nichols SA, Roberts BW, Richter DJ, Fairclough SR, and King N. 2012. Origin of metazoan cadherin diversity and the antiquity of the classical cadherin/β-catenin complex. *Proceedings of the National Academy of Sciences* 109:13046-13051. DOI: 10.1073/pnas.1120685109

Putnam NH, Butts T, Ferrier DE, Furlong RF, Hellsten U, Kawashima T, Robinson-Rechavi M, Shoguchi E, Terry A, and Yu J-K. 2008. The amphioxus genome and the evolution of the chordate karyotype. *Nature* 453:1064-1071. DOI: 10.1038/nature06967

Putnam NH, Srivastava M, Hellsten U, Dirks B, Chapman J, Salamov A, Terry A, Shapiro H, Lindquist E, Kapitonov VV, Jurka J, Genikhovich G, Grigoriev IV, Lucas SM, Steele RE, Finnerty JR, Technau U, Martindale MQ, and Rokhsar DS. 2007. Sea anemone genome reveals ancestral eumetazoan gene repertoire and genomic organization. *Science* 317:86-94. DOI: 10.1126/science.1139158

Ryan JF, Pang K, Schnitzler CE, Nguyen A-D, Moreland RT, Simmons DK, Koch BJ, Francis WR, Havlak P, and Smith SA. 2013. The genome of the ctenophore *Mnemiopsis leidyi*

and its implications for cell type evolution. *Science* 342:1242592. DOI: 10.1126/science.1242592

Shinzato C, Shoguchi E, Kawashima T, Hamada M, Hisata K, Tanaka M, Fujie M, Fujiwara M, Koyanagi R, Ikuta T, Fujiyama A, Miller DJ, and Satoh N. 2011. Using the *Acropora digitifera* genome to understand coral responses to environmental change. *Nature* 476:320-323. DOI: 10.1038/nature10249

Srivastava M, Begovic E, Chapman J, Putnam NH, Hellsten U, Kawashima T, Kuo A, Mitros T, Salamov A, and Carpenter ML. 2008. The *Trichoplax* genome and the nature of placozoans. *Nature* 454:955-960. DOI: 10.1038/nature07191

Srivastava M, Simakov O, Chapman J, Fahey B, Gauthier ME, Mitros T, Richards GS, Conaco C, Dacre M, and Hellsten U. 2010. The *Amphimedon queenslandica* genome and the evolution of animal complexity. *Nature* 466:720-726. DOI: 10.1038/nature09201

Stein L, Sternberg P, Durbin R, Thierry-Mieg J, and Spieth J. 2001. WormBase: network access to the genome and biology of *Caenorhabditis elegans*. *Nucleic Acids Research* 29:82-86. DOI: 10.1093/nar/29.1.82
